# The association between benzodiazepine use and greater risk of neurocognitive impairment is moderated by medical burden in people with HIV

**DOI:** 10.1007/s13365-022-01076-1

**Published:** 2022-04-07

**Authors:** Erin E. Sundermann, Rowan Saloner, Anna Rubtsova, Annie L. Nguyen, Scott Letendre, Raeanne C. Moore, Mariana Cherner, Qing Ma, María J. Marquine

**Affiliations:** 1 Department of Psychiatry, University of California, San Diego, 220 Dickinson St # B, San Diego, CA 92103 USA; 2 San Diego State University/University of California, San Diego Joint Doctoral Program in Clinical Psychology, San Diego, CA USA; 3 Department of Neurology, University of California, San Francisco, 505 Parnassus Ave, San Francisco, CA 94143 USA; 4 Department of Behavioral, Social, and Health Education Sciences, Emory University Rollins School of Public Health, 1518 Clifton Rd, Atlanta, GA 30322 USA; 5 Department of Family Medicine, Keck School of Medicine, University of Southern California, 1975 Zonal Ave, Los Angeles, CA 90033 USA; 6 Department of Medicine, University of California, San Diego, 220 Dickinson St # B, San Diego, CA 92103 USA; 7 Department of Pharmacy Practice, University of Buffalo, 285 Pharmacy Building Buffalo, New York, NY 14214 USA

**Keywords:** Benzodiazepines, HIV, Neurocognitive impairment, Comorbidities, Medical burden

## Abstract

Benzodiazepine use is linked to neurocognitive impairment (NCI) in the general population and people with HIV (PWH); however, this relationship may depend on age-related factors such as medical comorbidities, which occur at an elevated rate and manifest earlier in PWH. We retrospectively examined whether chronological age or medical burden, a clinical marker for aging, moderated the relationship between benzodiazepine use and NCI in PWH. Participants were 435 PWH on antiretroviral therapy who underwent neurocognitive and medical evaluations, including self-reported current benzodiazepine use. A medical burden index score (proportion of accumulated multisystem deficits) was calculated from 28 medical deficits. Demographically corrected cognitive deficit scores from 15 neuropsychological tests were used to calculate global and domain-specific NCI based on established cut-offs. Logistic regressions separately modeled global and domain-specific NCI as a function of benzodiazepine x age and benzodiazepine x medical burden interactions, adjusting for current affective symptoms and HIV disease characteristics. A statistically significant benzodiazepine x medical burden interaction (*p* = .006) revealed that current benzodiazepine use increased odds of global NCI only among those who had a high medical burden (index score > 0.3 as indicated by the Johnson–Neyman analysis), which was driven by the domains of processing speed, motor, and verbal fluency. No age x benzodiazepine interactive effects on NCI were present. Findings suggest that the relationship between BZD use and NCI among PWH is specific to those with greater medical burden, which may be a greater risk factor for BZD-related NCI than chronological age.

**Supplementary Information:**

The online version contains supplementary material available at 10.1007/s13365-022-01076-1.

## Introduction

Despite advances in effective antiretroviral therapies (ART), 45% of people with HIV (PWH) continue to experience neurocognitive impairment (NCI) (Heaton et al. 2010; Wei et al. 2020). These deficits among PWH are associated with complications in everyday function (e.g., unemployment, and poor medication adherence) (Woods et al. 2008; Laverick et al. 2017; Marquine et al. 2018) making it important to identify factors that render PWH vulnerable to NCI. One factor may be elevated rates of medical and psychiatric conditions among PWH and the extensive use of non-ART medications to treat these conditions. Benzodiazepines, the most frequently prescribed medication for anxiety and sleep disorders, have been implicated in cognitive deficits in the general population. Although not consistently (Fastbom et al. 1998; Verdoux et al. 2005; Zhang et al. 2016; Nader and Gowing 2020), epidemiological studies suggest that benzodiazepine use is a risk factor for NCI and/or cognitive decline (Barker et al. 2004; Stewart 2005; Wright et al. 2009; Dell’Osso et al. 2015; Picton et al. 2018). Benzodiazepine use, particularly long-term use (at-least 6 months), is also associated with elevated dementia risk (Lagnaoui et al. 2002; Wu et al. 2009, 2011; Boeuf-Cazou et al. 2011; Billioti de Gage et al. 2012, 2014; Gallacher et al. 2012).

These adverse effects of benzodiazepines may be particularly concerning among individuals with health conditions characterized by neurobehavioral dysfunction such as HIV. Our group previously found that, among PWH, benzodiazepine users had a higher risk for NCI than benzodiazepine non-users even with adjustment for demographics, symptoms of affective distress, clinical comorbidities (e.g., hypertension and diabetes), and HIV-related disease factors (e.g., nadir CD4) (Saloner et al. 2019b). These adverse effects of benzodiazepine use were driven by the domains of processing speed, motor function, executive function, and memory suggesting effects of benzodiazepines on neurocognitive slowing and deficits in memory and higher-order cognitive capacities. These findings hold great public health significance given higher rates of benzodiazepine use among PWH (24%) compared to the general population (~ 13%) (Wixson and Brouwer 2014), likely due to the higher prevalence of anxiety and sleep disorders in PWH (Taibi 2013; Brandt et al. 2017).

However, an association between benzodiazepine use and NCI has not been found consistently in the general population (Fastbom et al. 1998; Billioti de Gage et al. 2014; Zhang et al. 2016; Nader and Gowing 2020). Discrepancies may be due to sample and methodological differences across studies and/or the potential moderating role of demographic or clinical characteristics. In our prior study, the benzodiazepine use and NCI link among PWH was not ubiquitous with 25% of benzodiazepine users not demonstrating NCI (Saloner et al. 2019b). Age potentially moderates the benzodiazepines and NCI relationship given that age-related changes in pharmacokinetics and pharmacodynamics can increase sensitivity to drug effects on the central nervous system (Kruse 1990; Hämmerlein et al. 1998; Turnheim 2003). Considering the aging demographic of PWH (Centers of Disease Control and Prevention 2020) and the elevated rates (Schouten et al. 2014) and earlier onset (De Francesco et al. 2020) of age-related comorbidities among PWH, a moderating role of age may be particularly important in the context of HIV. However, aging is a highly heterogeneous process, especially among PWH where different aging phenotypes are influenced by sociocultural, genetic, medication, comorbidities and inflammation factors (Stoff et al. 2017). Thus, clinical (e.g., medical burden and frailty index) and biological (e.g., telomere length and epigenetic clock) markers of aging are likely better indicators of the aging process than chronological age.

We explored the association between benzodiazepine and NCI among PWH by examining the moderating role of chronological age and medical burden, as an index of biological age, in a community-based sample of ART-treated, virally suppressed PWH. Medical burden was operationalized as an index representing the accumulation of multisystem health deficits that are both HIV-related and non-HIV-related (e.g., diabetes, anemia, and renal dysfunction). This medical burden index, in different adaptations, has been associated with cognition, everyday function, and mortality among PWH (Guaraldi et al. 2015; Oppenheim et al. 2018; Paolillo et al. 2019). We hypothesized that older chronological age and greater medical burden would separately exacerbate the adverse effect of benzodiazepine use on NCI, but medical burden would have a stronger moderating role than chronological age. In secondary analysis, we examined whether the moderating role of chronological age and/or medical burden on the benzodiazepine and NCI link was domain specific.

## Methods

### Participants

Participants included 435 PWH enrolled in various NIH-funded observational studies within the HIV Neurobehavioral Research Program (HNRP, https://hnrp.hivresearch.ucsd.edu/) at the University of California San Diego (UCSD). Study details have been published elsewhere (Heaton et al. 1995, 2010; Morgello et al. 2001). Our deidentified data are available to qualified external investigators upon requests submitted to hnrpresource@ucsd.edu. Exclusion criteria for the parent studies included history of non-HIV-related neurological, medical or psychiatric disorders that affect brain function (e.g., diabetes), learning disabilities, dementia diagnosis, and English not a primary language. Our inclusion criteria were on ART with successful viral suppression (HIV-1 RNA < 50 copies/ml) and availability of variables of interest and covariates. Our exclusion criteria were positive urine toxicology for illicit drugs (except marijuana) or Breathalyzer test for alcohol during study visits. Medication use including benzodiazepines and estimated duration of use (missing in n = 15) were assessed via structured, clinician-administered questionnaires. Self-report of medication use was confirmed via review of medical regimen records if available. Eighty-one participants reported active use of prescribed benzodiazepines (benzodiazepine +), and 354 participants reported no use of prescribed benzodiazepines (benzodiazepine-). The following agents were reported by participants in the benzodiazepine + group: alprazolam (n = 19), clonazepam (n = 23), diazepam (n = 6), lorazepam (n = 29), temazepam (n = 8), estazolam (n = 1). Benzodiazepine use was further categorized as long-acting (N = 30) if reported clonazepam, diazepam, or estazolam use or short-acting (N = 51) if reported alprazolam, lorazepam, temazepam use. If both short-acting and long-acting medication types were reported, the participant was categorized as a long-acting benzodiazepine user. If a participant reported use of multiple benzodiazepines (regardless of type), the longest duration was used in analyses.

As a covariate, we calculated the total number of currently prescribed medications excluding ART and benzodiazepines. Due to evidence of an effect of opioid, antipsychotic and anticholinergic medication use on cognition (Coupland et al. 2019; Cuesta et al. 2001; Dufort and Samaan 2021), we also specifically assessed use of these medication types and considered as covariates.

UCSD’s Human Research Protections Program approved all study procedures, and all participants provided written informed consent. Participants completed comprehensive neuromedical and neurobehavioral assessments and blood draw during study visits.

### Neuromedical evaluation

HIV infection was diagnosed by enzyme-linked immunosorbent assay (ELISA) with confirmation by Western blot or real time-polymerase chain reaction (RT-PCR). HIV disease characteristics were determined via a combination of self-report (e.g., estimated duration of HIV disease) and laboratory tests (e.g., CD4 + T-cell count). Nadir CD4 + T-cell count was the lowest lifetime value by self-report, study-obtained CD4 + T-cell count, or released medical records. CD4 + T-cell count was measured with flow cytometry. Plasma HIV-1 RNA level was measured by ultra-sensitive RT-PCR (Amplicor, Roche Diagnostic System) in a CLIA-certified clinical laboratory. HIV-1 RNA was considered undetectable below the lower limit of quantitation of 50 copies/mL. Hepatitis C serostatus was measured via MedMira Multiplo rapid test (MedMira Inc.).

### Medical burden index

Medical burden was quantified based on previously established methods for constructing a frailty index in PWH (Guaraldi et al. 2015; Oppenheim et al. 2018). Here, the medical burden index was calculated as the proportion of health deficits from a group of 28 health variables, including clinical laboratory assays, medical comorbidities, and HIV-specific characteristics. Each variable was coded as “1,” when a deficit was present and “0,” when absent (see Table 1, Supplemental Digital Content, which displays the medical burden index components and their criteria). Thus, medical burden scores ranged from 0 (no deficits) to 1 (all deficits). The 28 selected variables were chosen based on all available neuromedical data from the parent studies that were consistent with variables included in prior indices (Guaraldi et al. 2015; Oppenheim et al. 2018). Consistent with published guidelines for creating a frailty index (Searle et al. 2008), we excluded factors that (1) had greater than 5% missing data (i.e., unintentional weight loss, LDL cholesterol) and (2) had less than 1% of participants meeting criteria for the deficit (i.e., abnormal sodium and peripheral vascular disease).

**Table 1:** Sample characteristics by benzodiazepine use group

	**Benzodiazepine- (n = 354)**	**Benzodiazepine + (n = 81)**	**p-value**
***Demographic factors***			
Age, *M (SD)*	51.7 (12.4)	56.6 (9.4)	.001
Years of education, *M (SD)*	13.9 (2.8)	14.4 (2.5)	.14
Race/ethnicity			.05
Non-Hispanic White, n (%)	189 (53.3%)	58 (71.6%)	
Hispanic, n (%)	102 (28.8%)	15 (18.5%)	
Black/African-American, n (%)	50 (14.1%)	5 (6.2%)	
Asian, n (%)	4 (1.1%)	1 (1.2%)	
Other, n (%)	9 (2.5%)	2 (2.5%)	
Male sex, n (%)	308 (87.0%)	71 (87.6%)	.88
***Clinical factors***			
Beck Depression Inventory-II score, *M (SD)*	9.7 (9.9)	14.1 (10.7)	.001
POMS Tension/Anxiety Subscale score, *M (SD)*^*a*^	9.1 (6.9)	11.6 (8.0)	.007
Current major depressive disorder, n (%)	24 (7.0%)	11 (14.3%)	.04
History of major depressive disorder, n (%)	184 (53.0%)	55 (67.9%)	.01
History of generalized anxiety disorder, n (%)	10 (2.8%)	4 (5.0%)	.57
Current antidepressant use, n (%)	136 (38.4%)	57 (74.0%)	< .001
Current illicit substance diagnosis^b^, n (%)	14 (4.1%)	2 (2.6%)	.53
History of illicit substance diagnosis^c^, n (%)	142 (40.9%)	39 (48.7%)	.20
Current alcohol use disorder^b^, n (%)	9 (2.6%)	2 (2.6%)	.87
History of alcohol use disorder^c^, n (%)	183 (52.7%)	37 (46.2%)	.52
Current cannabis use disorder^b^, n (%)	6 (1.7%)	3 (3.8%)	.44
History of cannabis use disorder^c^, n (%)	100 (28.8%)	20 (25.0%)	.71
Number of current medications (excluding ART and benzodiazepines), *M* (SD)	6.6 (6.2)	10.5 (7.7)	< .001
Current opioid medication use	29 (8.2%)	6 (18.2%)	.03
Current anticholinergic medication use	0 (0%)	0 (0%)	-
Current antipsychotic medication use	9 (2.5%)	3 (3.8%)	.02
NCI, n (%)	121 (35.5%)	37 (48.0%)	.04
Medical burden, *M* (SD)	0.25 (0.10)	0.28 (0.09)	.01
***HIV Disease Characteristics***			
Nadir CD4 + T-cells (/μl), *M (SD)*	218.4 (208.4)	173.5 (178.1)	.07
CD4 + T-cells (/μl), *M (SD)*	617.1 (285.9)	664.2 (316.2)	.19
Estimated duration of HIV disease (years), *M* (SD)	17.3 (9.6)	21.8 (9.3)	< .001
AIDS diagnosis, n (%)	216 (61.0%)	57 (70.4%)	.22

*MDD* major depressive disorder, *NCI* neurocognitive impairment *ART* antiretroviral therapy

^a^Data missing in 68 participants

^b^Data missing in 17 participants

^c^Data missing in 8 participants

### Neuropsychological evaluation

Participants completed a standardized neurocognitive test battery of verbal fluency, working memory, speed of information processing, learning and delayed recall, executive function, and complex motor function. Specific tests are described elsewhere (Cysique et al. 2011). Raw test scores were transformed into demographically adjusted (i.e., age, education, sex, and race/ethnicity) T-scores based on normative samples of HIV participants (Heaton et al. 2004; Norman et al. 2011). T-scores for each test were also converted into deficit scores that ranged from 0 (T-score ≥ 40, no impairment) to 5 (T-score < 20, severe impairment) and then averaged across all tests to obtain a global deficit score (GDS) and within domains to obtain domain deficit scores (DDS) (Heaton et al. 1995; Carey et al. 2004; Blackstone et al. 2012). Based on pre-established cut-points, global NCI was defined as GDS ≥ 0.50 and domain-specific NCI was defined as DDS > 0.50 (Carey et al. 2004).

### Neuropsychiatric evaluation

DSM-IV diagnoses of current and lifetime alcohol, cannabis, and illicit (amphetamine, cocaine, hallucinogens, inhalant, sedatives, opioids, and PCP) substance use disorders, major depressive disorder (MDD), and generalized anxiety disorder (GAD) were determined based on the fully structured computer-based Composite International Diagnostic Interview version 2.1 (World Health Organization 1997). The Beck Depression Inventory-version 2 (BDI-II) (Beck et al. 1996) was used to assess depressive symptoms within the past two weeks with higher scores reflecting greater affective symptoms. The 9-item tension/anxiety subscale of the Profile of Mood States (POMS; n = 367) (McNair et al. 1981) was administered to a subset of participants as a measure of anxious and nervous feeling in the past week. Given that BDI-II data were available on more participants and strongly correlated with the POMS tension/anxiety subscale (R = 0.71, *p* < 0.001), BDI-II was chosen to model affective distress in the primary statistical models; however, scores on the POMS tension/anxiety subscale were adjusted for in sensitivity analyses to see if results changed.

### Statistical analyses

Differences in sample characteristics between benzodiazepine groups were examined using Chi-square tests for categorical variables, t-tests for normally distributed continuous variables, and Kruskal–Wallis one-way analyses of variance (ANOVA) for non-normally distributed continuous variables as determined by the Shapiro–Wilk test. In order to demonstrate the utility of separately examining the moderating roles of chronological age and medical burden, we examined the relationship between chronological age and medical burden index. Then, using multivariable logistic regression, we examined the benzodiazepine use X age and benzodiazepine use X medical burden interaction terms on odds of NCI. Significant interactions were probed by a Johnson–Neyman analysis (Johnson and Neyman 1936; Preacher et al. 2006) to empirically identify the specific range of values of the moderator (i.e., age, medical burden, or both) for which the difference in odds of NCI between benzodiazepine groups was statistically significant. Interaction terms that were not significant were removed from the model in order to examine the main effects. Demographics and relevant clinical factors that were not included in the medical burden index were considered as covariates, *i.e.*, BDI-II score, history of MDD, history of GAD, current use of antidepressant, opiate, antipsychotic and anticholinergic medications, number of current medications (excluding benzodiazepines and ART), and current and past substance use disorders including alcohol, cannabis, cocaine, hallucinogens, inhalants, methamphetamine, opioids, PCP, sedatives, and others. Covariates that related to NCI at *p* ≤ 0.10 in univariate analyses were included in statistical models and were retained if significant in the multivariable model at *p* ≤ 0.10.

Upon detection of a significant relationship between benzodiazepine use and odds of NCI, either independently or through interactions with medical burden and/or age, we further probed the relationship by examining the following: (a) whether it was driven by long-acting versus short-acting benzodiazepines, (b) whether it was driven by specific cognitive domains, and (c) the relationship between duration of benzodiazepine use and odds of NCI among benzodiazepine users in order to test for a potential dose-dependent relationship.

Analyses were performed using SPSS 24 (SPSS Inc., Chicago, IL). Significance was defined as *p* ≤ 0.05 (two-sided) unless otherwise noted.

## Results

### Sample characteristics

**Fig. 1:**
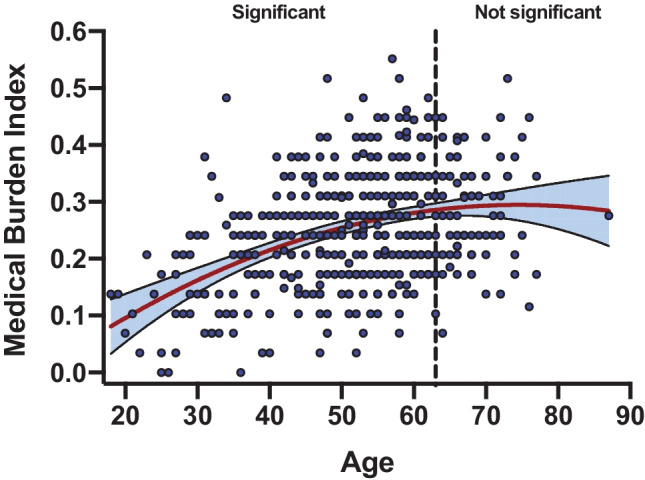
Region of significance analysis for the quadratic effect of age on medical burden index. Age exhibits a significant positive association with medical burden index until **age 63**, at which point the slope flattens and the relationship between age and medical burden is no longer significant

The sample of 435 PWH was 87% male and 50% non-Hispanic White, 27% Hispanic with a mean age of 52.6 (SD = 12.0; age range: 18–87) and mean years of education of 14.0 (SD = 2.7). Among benzodiazepine users, estimated duration of use ranged from 2–212 months with a median of 47 months. Sample characteristics by benzodiazepine status are displayed in Table 1. Compared to benzodiazepine non-users, benzodiazepine users were older and had a higher proportion of non-Hispanic Whites. As expected, benzodiazepine users had more depressive and tension/anxiety symptoms, a higher prevalence of current and past MDD diagnoses, a higher prevalence of opioid and antipsychotic medication use and higher medical burden index scores compared to benzodiazepine non-users. In contrast, benzodiazepine groups were comparable in rates of current and past substance use disorders and in HIV disease characteristics with the exception of a longer duration of HIV disease in benzodiazepine users versus non-users. Consistent with our previous results in a sample of PWH with and without viral suppression (Saloner et al. 2019b), we found higher rates of NCI in benzodiazepine users (49.3%) versus non-users (36.1%) (*X*^2^ = 4.86, *p* = 0.03) among ART-treated, virally suppressed PWH. Among considered covariates, BDI-II scores, lifetime MDD diagnosis, number of medications, antidepressant and antipsychotic medication use and current alcohol use disorder were associated with NCI at *p* ≤ 0.10 and, thus, were included in all statistical models. When examining the relationship between chronological age and medical burden index, we found a significant quadratic relationship (*p* = 0.004) whereby age positively related to medical burden index until age 63, as determined by the Johnson–Neyman analysis, at which point this relationship is nonsignificant (Fig. 1).

### Moderating role of chronological age and medical burden in the benzodiazepine and NCI link

**Fig. 2:**
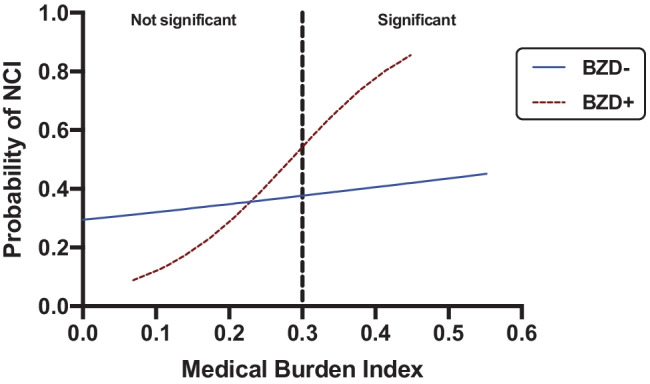
Johnson–Neyman region of significance analysis indicating an adverse effect of benzodiazepine use on NCI only when medical burden index is ≥ 0.3. NCI = neurocognitive impairment. BZD- = benzodiazepine non-user. BZD +  = benzodiazepine user

Among included covariates (BDI-II scores, history of MDD, history of GAD, current alcohol use disorder, number of medications), BDI-II scores and antipsychotic medications were retained in final statistical models due to its significance in the multivariable models (*p* ≤ 0.10). The benzodiazepine use X age interaction was not significant indicating no moderating role of age in the adverse effect of benzodiazepine use. There was also no main effect of age on NCI. In contrast, we detected significant benzodiazepine use X medical burden interactive effects on NCI (Table 2). In probing this interaction, the Johnson–Neyman analysis revealed that the adverse effect of benzodiazepine use on the odds of NCI is only observed when the medical burden index is greater than 0.3 reflecting presence of at-least 30% of the considered health deficits (Fig. 2). Among those with medical burden ≥ 0.3, benzodiazepine use related to an almost threefold increased likelihood of NCI (OR = 2.76, 95%CI = 1.23–6.19, *p* = 0.01; Fig. 2). However, benzodiazepine use did not relate to NCI among those with medical burden ≤ 0.3 (OR = 0.90, 95%CI = 0.45–1.80, *p* = 0.76). When adjusting for POMS tension/anxiety scores in sensitivity analysis in a participant subset (N = 367), results did not change except that the association between benzodiazepine use and higher odds of NCI was slightly attenuated in the higher medical burden group (OR = 2.51, 95%CI = 1.08–5.83, p = 0.03).

**Table 2:** The multivariable logistic regression model predicting global NCI

**Predictor**	**B (SE)**	**p-value**	**OR**	**95% CI**
Medical burden X benzodiazepine use	0.90 (0.34)	**.009**	2.45	1.25–4.81
Age	0.007 (0.01)	.42	1.01	0.99–1.03
Medical burden index^a^	0.09 (0.12)	.46	1.09	0.87–1.37
benzodiazepine use (vs. no benzodiazepine use)^b^	-2.13 (1.00)	**.03**	0.12	0.02–0.84

Analyses adjusted for BDI-II scores and antipsychotic medications. The age X benzodiazepine use interaction terms was not significant (B = 0.02, SE = 0.03, p = .50), so was removed from the model

^a^Due to the interaction effect, this lower-order term represents the effect of medical burden index on NCI in the no benzodiazepine use reference group

^b^Due to the interaction effect, this lower-order term represents the effect of benzodiazepine use on NCI when medical burden index is average

We next examined whether our findings were driven by long-acting versus short-acting benzodiazepines. Although the interaction of medical burden with either long-acting (OR:2.64, 95%CI:0.83–8.39, p = 0.10) or short-acting (OR:1.66, 95%CI:0.86–3.22, p = 0.13) benzodiazepines was not significant, both use of both types of benzodiazepines (compared to no benzodiazepine use) related to a higher odds of NCI only within those with a medical burden index ≥ 0.3 (long-acting: OR:2.83, 95%CI:0.84–9.48, p = 0.09; short-acting: 2.86, 95%CI:1.15–7.15, p = 0.02). Although this relationship was the same magnitude for short-acting and long-acting benzodiazepines, it was only significant for the short-acting type likely due to the larger sample and, thus, greater statistical power. When examining duration of benzodiazepine use among benzodiazepine users, we found no relationship between the benzodiazepine X medical burden interaction and odds of NCI (OR = 1.10, 95%CI = 0.95–1.26, p = 0.19). There was also no relationship between duration of benzodiazepine use and odds of NCI among those with high medical burden (index ≤ 0.3) (OR = 1.00, 95%CI = 0.92–1.09, p = 0.97); however, although benzodiazepine duration ranged from 2 to 212 months, variability was limited in that the majority of users (70%) reported a duration within about 3–5 years.

### Cognitive domain-specific analyses

Significant medical burden X benzodiazepine interactions were observed for speed of information processing, verbal fluency and motor impairment (Table 3). The Johnson–Neyman analysis revealed that the adverse effect of benzodiazepine use on the odds of NCI is only observed when the medical burden index is greater than or equal to 0.32 for speed of information processing, 0.34 for motor and 0.27 for verbal fluency. These interactions revealed a significantly higher likelihood of speed of information processing (OR = 3.79, 95%CI = 1.06–13.53, *p* = 0.04), motor (OR = 3.34, 95%CI = 1.24–8.99, *p* = 0.02) and verbal fluency (OR = 2.58, 95%CI = 1.28–5.22, *p* = 0.008) impairment in benzodiazepine users versus non-users only among those above the respective Johnson–Neyman-derived cut-point of the medical burden index. Benzodiazepine use independently related to a higher likelihood of learning and recall impairment and working memory/attention impairment. When adjusting for POMS tension/anxiety scores in sensitivity analysis in a participant subset (N = 367), the relationships between benzodiazepine use and higher odds of processing speed (OR:2.52, 95%CI:0.63–10.1, p = 0.19), motor (OR:3.09, 95%CI:1.10–8.65, p = 0.03), and verbal fluency (OR:2.38, 95%CI:1.12–5.03, p = 0.02) impairment were attenuated although remained significant except for processing speed. Results for all cognitive domains did not change substantively when adjusting for POMS tension/anxiety scores.

**Table 3:** Results from multivariable logistic regression models examining the separate and interactive associations of benzodiazepine use and medical burden index with domain-specific NCI

**Domain/predictor**	**B (SE)**	**p-value**	**OR**	**95% CI**
**Verbal fluency**				
Medical burden X benzodiazepine use	0.73 (0.33)	**.03**	2.07	1.08–3.94
Benzodiazepine use (vs. no benzodiazepine use)^b^	-1.07 (0.92)	**.**24	0.34	0.06–2.08
Medical burden index^a^	0.04 (0.12)	.72	1.04	0.82–1.33
**Executive function**				
Medical burden X benzodiazepine use	-	-	-	-
Benzodiazepine use (vs. no benzodiazepine use)	0.30 (0.27)	.26	1.35	0.80–2.27
Medical burden index	0.09 (0.11)	.40	1.10	0.88–1.37
**SIP**				
Medical burden X benzodiazepine use	0.93 (0.38)	**.01**	2.54	1.21–5.33
benzodiazepine use (vs. no benzodiazepine use)^b^	-2.51 (1.15)	**.03**	0.08	0.01–0.77
Medical burden index^a^	0.06 (0.13)	.68	1.06	0.81–1.37
**Learning**				
Medical burden X benzodiazepine use	-	-	-	-
Benzodiazepine use (vs. no benzodiazepine use)	0.49 (0.26)	**.05**	1.64	0.99–2.70
Medical burden index	-0.01 (0.11)	.91	0.99	0.80–1.22
**Recall**				
Medical burden X benzodiazepine use	-	-	-	-
Benzodiazepine use (vs. no benzodiazepine use)	0.83 (0.26)	**.001**	2.30	1.39–3.82
Medical burden index	0.05 (0.11)	.62	1.06	0.85–1.31
**Working memory/attention**				
Medical burden X benzodiazepine use	-	-	-	-
Benzodiazepine use (vs. no benzodiazepine use)	0.53 (0.27)	**.046**	1.70	1.01–2.87
Medical burden index	0.02 (0.12)	.89	1.02	0.81–1.28
**Motor**				
Medical burden X benzodiazepine use	0.76 (0.37)	**.04**	2.14	1.04–4.40
Benzodiazepine use (vs. no benzodiazepine use)^b^	-2.03 (1.12)	.07	0.13	0.01–1.17
Medical burden index^a^	0.18 (0.13)	.17	1.20	0.92–1.55

Analyses adjusted for BDI-II scores and antipsychotic medications. When the medical burden X benzodiazepine use interaction term was not significant, it was removed from the final model when assessing main effects.

*NCI *neurocognitive impairment, *SPI* speed of information processing

^a^Due to the interaction effect, this lower-order term represents the effect of medical burden index on domain-specific impairment in the no benzodiazepine use reference group

^b^Due to the interaction effect, this lower-order term represents the effect of benzodiazepine use on domain-specific impairment when medical burden index is average

## Discussion

In a virally suppressed sample of PWH, we extend previous finding of a relationship between benzodiazepine use and a higher likelihood of NCI by showing that this relationship depends on medical burden. Only among PWH with a medical burden index greater than 0.3 did benzodiazepine use relate to a higher likelihood of NCI. Conversely, the association between benzodiazepine use and cognitive function was not modified by chronological age. In domain-specific analyses, these results appeared to be driven by executive function, speed of information processing, motor and verbal fluency.

Medical burden, an indicator of biological age, but not chronological age moderated the relationship between benzodiazepine use and NCI in PWH. Although medical burden is often age-related, it is characteristically distinct from chronological age (Buchner and Wagner 1992; Fried et al. 2004). Similar to Oppenheim et al (2018) (Oppenheim et al. 2018), we found a quadratic relationship whereby age positively related to medical burden only before age 63. Medical burden is postulated to reflect physiological or biological reserve more than chronological age in PWH given that age-related conditions and geriatric syndromes (e.g., frailty and dementia) are found in relatively young PWH (Brew et al. 2005; Soontornniyomkij et al. 2012; So-Armah et al. 2014; Greene et al. 2017), whereas a sizable proportion of older PWH experience no declines in cognitive or physical functioning (Saloner et al. 2019a; Fazeli et al. 2020). Therefore, the profile of health captured by the medical burden index may optimally represent biological age. Supporting this notion were findings from a study by Levine et al. (Levine et al. 2016) that created a measure of age acceleration based on the difference between biological aging, defined by DNA methylation levels, and chronological age in postmortem brain tissue of PWH. Greater age acceleration, but not chronological age, related to a higher likelihood of pre-mortem NCI. In the multisite CHARTER cohort, chronological age was strongly associated with poorer white matter integrity and less subcortical gray matter in PWH with severe comorbidity burden, yet this association was attenuated in PWH with minimal-to-moderate comorbidity burden (Saloner et al. 2019c).

When probing and benzodiazepine and NCI relationship, we found that both short- and long-acting BZDs related showed a relationship with higher odds of NCI that was of a similar magnitude although this relationship was only significant for short-acting benzodiazepines likely due to the larger cell size (n = 51 vs. 30). The consistency in findings between short- and long-acting benzodiazepines is similar to other studies reporting a relationship between benzodiazepine use and higher risk of dementia in the general population that did not differ between short- and long-acting substances (Gomm et al. 2016; Billioti de Gage et al. 2014); however, others have reported a stronger links between long-acting versus short-acting benzodiazepine use and cognitive impairment (Picton et al. 2018; Aldaz et al. 2021). Discrepancies are likely due to sample and methodological differences across studies and/or the potential confounding effects of other benzodiazepine use characteristics or clinical characteristics. Due to evidence in the literature of a stronger effect of benzodiazepine use on cognition with longer durations of use (Picton et al. 2018; Barker et al. 2004), it was unexpected that we did not observe a relationship between longer durations and odds of NCI among benzodiazepine users. However, this may be due to the limited variability in benzodiazepine use duration in our sample in that 70% of our sample reported a duration of 3–5 years. Given evidence that it is particularly long-term BZD use (at-least 1 year) that has adverse cognitive consequences (Nader and Gowing 2020; Barker et al. 2004; Crowe and Stranks 2018; Stewart 2005), the large proportion of long-term benzodiazepine use in our sample likely drove our findings.

Domain-specific analyses showed that the benzodiazepine use X medical burden interaction was evident in speed of information processing, verbal fluency and motor function. This is consistent with our prior study that also found an adverse effect of benzodiazepine use on speed of information processing and motor domains among PWH (Saloner et al. 2019b). A commonality among speed of information processing, motor function, and verbal fluency is that they are all speed-dependent and regulated by frontostriatal neural circuitry (Ances et al. 2012; Hakkers et al. 2017), which aligns with the theory that benzodiazepine causes psychomotor slowing (Rollings et al. 1994; Stewart 2005; Lader 2011). Frontostriatal circuity is also known to be disrupted in HIV (Du Plessis et al. 2014), which poses the question of potential compounding effects of benzodiazepine use and HIV infection on frontostriatal processing. In contrast, benzodiazepine use was independently associated with a higher likelihood of impairments in learning, recall and working memory/attention regardless of medical burden. This finding is also consistent with our prior findings that PWH who report benzodiazepine use and are positive for benzodiazepine in a toxicology test show working memory/attention and delayed recall impairments compared to PWH without evidence of benzodiazepine use (Saloner et al. 2019b). Similarly, a recent study from the Women’s Interagency HIV Study that reported an interaction between anxiolytic use (primarily benzodiazepines) and HIV serostatus on learning performance, whereby HIV-related learning deficits were only observed among those reporting anxiolytic use (Rubin et al. 2018). Our findings suggest that the adverse effects of benzodiazepine use on cognition may be more robust for the learning and memory domains given that it is observed consistently across studies and, in the current study, regardless of medical burden.

The underlying mechanisms and the temporal pattern of the benzodiazepine and NCI relationship are unclear. Insomnia and anxiety, the most common health conditions in which benzodiazepine are prescribed, can occur in the Alzheimer’s disease (AD) prodrome and, thus, the association may be an artifact of the use of benzodiazepines to treat early AD symptoms (Picton et al. 2018); however, suspected AD is currently rare among PWH (Morgello et al. 2018) given their shorter lifespan historically. Additionally, findings from longitudinal studies suggest a causative role of BZD on NCI including AD risk relating more strongly to long-term versus short-term benzodiazepine exposure (Billioti de Gage et al. 2014), and small improvements in memory performance among nursing home residents 4 weeks after benzodiazepine use was discontinued compared to residents that continued using benzodiazepines (Salzman et al. 1992).

There are potential reasons why benzodiazepine use is associated with NCI only in the context of high medical burden. It could suggest that the association between benzodiazepine use and NCI is more due to the cognitive effects of the underlying comorbidities rather than benzodiazepines themselves. However, we do not think this is likely because medical burden did not relate to NCI in benzodiazepine non-users as indicated by the nonsignificant medical burden term in our model including the medical burden X benzodiazepine interaction term. Another possibility is that multiple comorbidities could have cumulative effects on common underlying pathologies like chronic inflammation, hyperlipidemia and insulin resistance that weaken physiological reserve and heighten susceptibility to the effects of stressors; benzodiazepine in this case. HIV-related biological mechanisms and ART medications are associated with these common pathologies potentially making the cumulative effects of multimorbidity even more salient in HIV (D’Aversa et al. 2005; Deeks and Phillips 2009; Deeks 2011). Other measures of medical burden or physiological reserve including the Veterans Aging Cohort Study Index have been directly associated with NCI among PWH (Marquine et al. 2014, 2016; Zamudio-Rodríguez et al. 2018; Paolillo et al. 2020) suggesting that medical burden and benzodiazepine use may have compounding effects on NCI. Additionally, multimorbidity is typically coupled with polypharmacy, defined as more than 5 concurrent medications, which leads to higher risks of drug-drug and drug-disease interactions along with potentially cumulative toxicity. Although we covaried for antidepressant use and total number of medications, benzodiazepine may interact with specific medications resulting in compounding effects on brain health.

The clinical relevance of our findings is significant given the greater burden of comorbidities in PWH (Justice et al. 2012; Hosaka et al. 2019) and the higher rates of benzodiazepine use (Wixson and Brouwer 2014) versus the general population. Importantly, our sample of PWH were all on ART and virally suppressed indicating that the deleterious effects of combined benzodiazepine use and higher medical burden on NCI occur regardless of successfully treated HIV. Additionally, benzodiazepine use represents a modifiable risk factor. By identifying individuals at higher risk of benzodiazepine-related cognitive deficits, clinicians can prioritize alternative pharmaceutical or behavioral strategies to treating anxiety and insomnia in these patients. Lastly, our results suggest that medication use and medical burden may help explain some of the variability in cognitive function among PWH and more accurately characterize brain health than chronological age.

Our study has limitations. Assessment of benzodiazepine and some of the medical deficits were self-reported and thus subject to recall biases. Given that benzodiazepines are commonly prescribed for anxiety and sleep disorders, we would ideally adjust for these conditions. While were are able to adjust for GAD and anxiety/tension symptoms, we did not have data on sleep-related conditions. Our cross-sectional design precludes investigation of the temporal relationship between benzodiazepine use and NCI. Longitudinal studies are needed. Although it would be informative to compare individual types of benzodiazepine use in their relationship with NCI, we examined a combined group of all current benzodiazepine users in order to sustain adequate statistical power to test the interactive effects of benzodiazepine use, age, and medical burden. Given evidence in the general population that benzodiazepine effects on cognition are related to dose and duration (Barker et al. 2004; Crowe and Stranks 2018), the availability of these characteristics would have allowed us to more definitively test and characterize the benzodiazepine and NCI relationship. The absence of an HIV-uninfected comparison precluded us from determining whether our results are specific to the context of HIV. Study strengths include a large and neuropsychologically well-characterized sample of PWH and calculation of a comprehensive medical burden index of 28 health conditions that span multiple organ systems and show adverse health effects. Also, whereas studies typically contend with the confounding presence of comorbidities when assessing effects of medications on NCI, we directly modeled medical burden as a predictor/moderator.

## Conclusions

Our findings suggest that the negative impact of benzodiazepine use on cognitive function among PWH may be specific to or particularly worrisome for those with greater medical burden. The moderating role of medical burden in the benzodiazepine use and NCI link seems to be specific to domains associated with psychomotor slowing. Our findings of the unique moderating role of medical burden in the benzodiazepine and NCI relationship may potentially extend to the general population and help explain inconsistencies in the broader literature.

## Supplementary information

Below is the link to the electronic supplementary material.

Supplementary file1 (PDF 117 KB)
