# Supplementary material for: The association between benzodiazepine use and greater risk of neurocognitive impairment is moderated by medical burden in people with HIV
Source: J Neurovirol. 2022 Apr 7;28(3):410–21. doi: 10.1007/s13365-022-01076-1 (PMC9470605; doi:10.1007/s13365-022-01076-1)
Supplement: Supplementary file 1 — Supplementary file1 (PDF 117 KB) [file 13365_2022_1076_MOESM1_ESM.pdf]

Supplemental Table 1. Individual components of the medical burden index and each deficit criterion and mode of assessment.

Variable	Deficit Criteria	Mode of Assessment
Clinical Measurements		
1. Abnormal BMI	>25 or <18kg/m^2	In-clinic measure
2. Low white blood cell count	<4000 cells/µl	Laboratory value
3. Abnormal BUN	<8 mg/dl or >23 mg/dl	Laboratory value
4. Low albumin (serum)	<3.5 mg	Laboratory value
5. Elevated fibrinogen	>3.25	Laboratory value
6. Low eGFR	<60	Laboratory value
7. Low hemoglobin	Male: <12 µmol/l; Female: <10 µmol/l	Laboratory value
8. Elevated AST	>31 U/l	Laboratory value
9. Elevated ALT	>31 U/l	Laboratory value
10. Elevated triglycerides	≥150 mg/dl	Laboratory value
11. Elevated total cholesterol	>200 mg/dl	Laboratory value
12. Low HDL cholesterol	Male: <40 mg/dl; Female: <50 mg/dl	Laboratory value
13. Elevated glucose	>200 mg/dl	Laboratory value
14. Low platelets	<150 billion/l	Laboratory value
Comorbidities		
15. Cerebrovascular accident	Positive	Self-report/medical records
16. COPD	Positive	Self-report/medical records
17. Diabetes mellitus	Positive	Self-report/medical records
18. HCV	Positive	Self-report/medical records
19. Hyperlipidemia	Positive	Self-report/medical records
20. Hypertension	Positive or >130 mmHg systolic or > 85 mmHg diastolic	Self-report/medical records/In-clinic measure
21. Liver disease (mild)	Positive	Self-report/medical records
22. Malignancy	Positive	Self-report/medical records
23. Myocardial infarction	Positive	Self-report/medical records
24. Renal disease	Positive	Self-report/medical records
HIV Specific		
25. Low current CD4	<500 cells/µl	Laboratory value
26. Nadir CD4	<200 cells/µl	Laboratory value
27. CD4/8 ratio	<1.0	Laboratory value
28. Duration of infection	>10 years	Self-report

Notes. BMI = body mass index, BUN = blood urea nitrogen, eGFR = Estimated Glomerular Filtration Rate, AST = Aspartate transaminase, ALT = Alanine transaminase, HDL = high density lipoprotein, COPD = chronic obstructive pulmonary disease, HCV = hepatitis c virus
